# A manometry classification to assess pelvic floor muscle function in women

**DOI:** 10.1371/journal.pone.0187045

**Published:** 2017-10-30

**Authors:** Priscylla Helouyse Angelo, Larissa Ramalho Dantas Varella, Maria Clara Eugênia de Oliveira, Monayane Grazielly Leite Matias, Maria Aneilma Ribeiro de Azevedo, Luzinete Medeiros de Almeida, Paulo Roberto Medeiros de Azevedo, Maria Thereza Micussi

**Affiliations:** 1 Physiotherapy Department, Federal University of Rio Grande do Norte, Natal, Rio Grande do Norte, Brazil; 2 Statistics Department, Federal University of Rio Grande do Norte, Natal, Rio Grande do Norte, Brazil; Jorge|Ferreira, BRAZIL

## Abstract

**Objective:**

To develop a classification scale for manometry of pelvic floor muscles (PFM) in Brazilian women, according to the modified Oxford scale.

**Methods:**

A cross sectional study, with 288 women enrolled in the Maternity, Natal, Brazil. Manometry and PFM strength data were collected and classified according to the modified Oxford scale. A simple linear regression was performed to determine the classification scale of manometry using the modified Oxford scale as the explanatory variable and the arithmetic mean of the manometry measurements as the response variable.

**Results:**

The average age was 52.80 (±8.78; CI: 51.67–53.93) years. Manometry showed an average of 35.1 (±22.7; CI: 32.1–38.0) cmH_2_O and most women (29.7%) scored grade 3 on the modified Oxford scale. According to the proposed scale, values between 7.5 to 14.5 cmH_2_O correspond to very weak pressure; 14.6 to 26.5 cmH_2_O represent weak pressure; 26.6 to 41.5 cmH_2_O represent moderate pressure; 41.6 to 60.5 cmH_2_O represent good pressure, and values above 60.6 cmH_2_O correspond to strong pressure.

**Conclusion:**

Manometry values were rated on a five-point scale. It is possible to rank the pressure levels performed by voluntary contraction of PFM with this new scale.

## Introduction

Pelvic floor muscles (PFM) are a set of muscles responsible for supporting the pelvic organs and for maintaining continence [1,2]. The pelvic diaphragm, urogenital diaphragm and anal and urethral sphincters form the PFM [3]. The structures that make up the pelvic floor (PF) work as a unit and for this reason the anatomical–functional relationship is very important for the maintenance of normal PF function [4].

Lesions that occur to PFM arising from women's life events can lead to incontinence, constipation, decreased or lack of muscle strength and genital prolapse, which impact on quality of life [5]. For this reason, a proper assessment of the function, strength and integrity of PFM has a special role in diagnosing and treating disorders involving this region [6,7]. A strength assessment may give evidence of the status of muscle weakness severity in addition to being essential for designing specific exercise programs, as well as for monitoring rehabilitation progress [8]; there are several PFM assessment techniques for this [3].

PFM function can be assessed using several different methods, including digital palpation. This method is perhaps the most accessible, but its reproducibility is conflicting [9]. In contrast, the Oxford scale has been used for over 20 years [1]. This scale is widely used by physiotherapists, and for its correct use examiner’s experience is essential [7]. Muscle testing depends on the cooperation and position of the subject, and experience of the examiner [10].

In 1950, Kegel was the first to report data on manometry using a manometer for measuring intravaginal pressure [11]. Perineometers are intended to measure the pressure changes captured in the vagina in response to voluntary contraction of PFM [8,12]. They are simple, minimally invasive and low-cost instruments [8,12].

Manometry measurements obtained between two evaluators demonstrate a strong correlation between them [13]. It is important to mention that this occurs when evaluations are carried out on the same day. Hundley et al. [12] suggest that manometry can provide reliable and reproducible data regarding PFM strength.

In addition, manometric measures are recommended by the International Continence Society (ICS) to assess PFM function [14]. Despite manometry being a reliable method and widely used in clinical research [5], to the authors’ knowledge there are no literature reference values in manometry of the pelvic floor muscle, which affects interpreting the assessment results. Therefore, our objective was to develop a classification scale for manometry in Brazilian women, according to the modified Oxford scale.

## Materials and methods

### Design

An observational, cross sectional study developed at the Multiuser Laboratory of Clinical and Epidemiologic Research (PESQCLIN), Natal, Brazil.

### Participants

Participants provided written informed consent. The study was approved by the Federal University of Rio Grande do Norte Research Ethics Committee, and was conducted in accordance with the principles of the Declaration of Helsinki.

The sample was the result of a non-probability sampling process. Women were recruited by spontaneous demand in gynecology outpatient clinics and in the climacterium in the maternity, during the period from September 2015 to June 2016. The study included women aged 18 to 80 years without an intact hymen, with no urinary or vaginal infection, no gynecological bleeding, no neurological disorders that could affect cognitive ability, and who had not had deliveries or gynecological surgery performed for at least six months. Patients were excluded if they gave up or withdrew consent to participate, felt pain during the introduction of the vaginal probe, could not perform isolated contraction of PFM or without advanced genital prolapse which makes evaluation difficult or painful.

### Assessment

An assessment form developed by the researchers was used with information on patient identification, socioeconomic and demographic data, as well as clinical, gynecological and obstetrical history. Weight and height were determined by physical examination using a digital scale (Bioland EB9010, Brazil) and a stadiometer (Sanny—Standard ES2030, Brazil). Body mass index was calculated by dividing weight by height squared, and classified according to the World Health Organization (WHO) [15].

This study used a Peritron 9300V (Cardio Design, Australia). It has a vaginal probe of 26 mm in diameter and 108 mm in length, and an active measurement surface of 55 mm. During assessment, air captured by the vaginal probe is shifted through a connecting pipe to a pressure sensor on the display unit. The signal issued by the pressure sensor is displayed in centimeters of water (cmH_2_O). The unit's operating range varies from zero to 300 cmH_2_O. Varella et al. [13] using the Peritron obtained a strong correlation and high reliability between two examiners (ICC = 0.98). Pereira et al. [16] study used the same equipment and demonstrated a strong correlation between the value of three valid voluntary contractions of PFM during the evaluation (ICC = 0.97). In addition, Ferreira et al. [17] found a moderate inter-rater reliability for the Peritron manometer. A recent study [18] indicated a high inter-rater reliability of manometry (Lin´s Concordance Correlation Coefficient = 0.95).

To perform digital palpation and manometry, the volunteer remained in a gynecological position (the supine position) with bent knees, hips flexed and abducted, and was naked from the abdomen down. The patients were instructed on the correct way to contract PFM, dissociating from abdominal muscles, hip adductors and glutes. The volunteer was also instructed to breathe normally, avoiding the Valsalva maneuver, and to perform muscle contraction with the greatest strength possible. Volunteers were also instructed to empty their bladders before the PFM function assessment.

Digital palpation was initially performed to verify quantitatively and qualitatively the voluntary contraction of the PFM. The evaluator requested the volunteer to contract her muscles as hard as she could, according to previously given instructions [19]. To quantify PFM strength, the evaluator inserted the first two phalanges of the second and third fingers smeared in lubricant gel with a gloved hand into the anterior third of the vaginal opening and requested a maximal voluntary contraction by giving the command "squeeze my fingers". Next, muscle strength was classified according to the modified Oxford scale into: 0 (nil), 1 (flicker), 2 (weak), 3 (moderate), 4 (good) to 5 (strong) [20].

After four minutes of rest, manometry was performed [21]. The vaginal probe was covered with a non-lubricated latex condom. A lubricating gel was used to insert the proble into the vaginal cavity, with the equipment turned off. Three maximum voluntary contractions of PFM were requested, with two to three seconds of duration each [8]. The command was "squeeze the probe". There was an interval of 30 seconds of rest between the muscle contractions [22]. The device was reset to zero for each contraction. All PFM function assessments were performed by a single evaluator, a physiotherapist expert in PFM function assessment. This analysis considered the average of the three squeezes [16].

### Data analysis

The G Power program (version 3.1) was used to calculate the study power. The following values were entered into the software: regression slope (1.35), α/β ratio (1.0), sample size (259), standard deviation of the average of the three squeezes (22.7), and the mean of the sample score in the modified Oxford scale (1.17). A 99% study power was obtained.

The collected information was then tabulated in the IMB Statistical Package for Social Sciences (version 20.0) for Windows for descriptive analysis, in which the variables are presented as mean, standard deviation, confidence intervals and frequency. A simple linear regression was performed to determine the classification scale of manometry using the modified Oxford scale as the explanatory variable and the arithmetic mean of the manometric measurements as the response variable.

Linear regression was performed on the statistical program R version 3.2.4. The Shapiro-Wilk test analyzed the normality of the regression residuals.

## Results

288 women were included in the study: 8 were excluded for having zero degree of strength (no noticeable muscle contraction) on the modified Oxford scale, 7 for experiencing pain when the probe was introduced, and 14 for failing to dissociate PFM contraction from accessory muscles (abdominal, gluteal and adductor muscles), representing a rate of 10.06% total sample loss.

The final sample consisted of 259 women, whose data were analyzed. The mean age was 52.80 (±8.78; CI: 51.67–53.93) years; mean BMI was 28.70 (±4.75; CI: 28.00–29.31) kg/m^2^. The average number of pregnancies was 3.31 (±2.52; CI: 3.00–3.63), the average for normal delivery was 2.27 (±2.53; CI: 1.96–2.58), and the average for cesarean delivery was 0.59 (±0.87; CI: 0.48–0.70).

Regarding the PFM function assessment, manometry showed an average of 35.1 (±22.7; CI: 32.1–38.0) cmH_2_O. On the modified Oxford scale, 18.5% (48) women had a grade one strength, 27.7% (72) grade two strength, 29.7% (77) grade three, 16.2% (42) grade four and 7.7% (20) grade five.

The coefficient of determination obtained from the performed regression was 72.23%. Regression residual analysis showed that the error variance was constant and showed a normal distribution (p = 0.93). Point and interval estimates were obtained with 99% confidence from the regression results for the average variable response in the case of modified Oxford scale to assume its possible values.

Table 1 shows the point estimates and average manometry, by interval. The values were rounded so that their limits were more convenient. The upper and lower limits of the final categorization are based in the confidence interval, dividing the space between the upper limit of a category by the lower limit of the other category to be added to the margin.

**Table 1:** Average manometry prediction based on linear regression.

Modified Oxford scale	Point prediction of average manometry	CI^a^
Flicker	9.3	7.7–11.1
Weak	19.5	17.9–21.1
Moderate	33.3	31.4–35.2
Good	50.8	47.4–54.3
Strong	72.0	66.1–78.2

^a^: 99% confidence interval.

Thus, the categorization of manometric measurements was performed on a five-point scale, ranging from a very weak pressure to a high pressure (Table 2).

**Table 2:** Classification scale of manometry.

Classification	Manometry values
Very weak	7.5–14.5
Weak	14.6–26.5
Moderate	26.6–41.5
Good	41.6–60.5
Strong	> 60.6

## Discussion

Scientific studies require assessment tools that are reliable so that their data can be used in clinical practice. In this sense, the perineometer is an instrument that has high reliability for strength measurements and PFM resistance [8]. However, manometric values have not yet been classified in clinical practice.

This study used the modified Oxford scale as the basis for a new classification of measurements obtained by manometry. Literature shows that the Oxford scale is often used in clinical practice and has good agreement with the Peritron (0.73) [23]. Kerschan-Schindl et al. [24] report a high correlation between the Peritron measurements and digital assessment of strength (r > 0.7) in a small group of 37 elderly women with UI. Sartore et al. [25] found a moderate agreement between the Oxford scale and vaginal pressure measures with a perineometer (k = 0.47). This variation can be due to the different methodologies applied on the studies such as sample (size, women group) or method of evaluating the PFM.

In addition to bidigital palpation, other PFM assessment methods also have good agreement with manometry. In the study by Chehrehrazi et al. [26], manometry presented a high correlation with transabdominal ultrasound measurements. The study by Pereira et al. [27] found a moderate correlation between manometry and electromyography. Both studies were performed with the Peritron.

Our study did not consider the factors of age, parity and hormonal status, since the aim was not to evaluate the relationship of these factors with the degree of PFM strength. However, literature shows that manometric measurements vary in women, according to the delivery type and the reported race [21]. It has also been shown that increased waist circumference is associated with decreased manometric values [28].

However, results of other studies point that parity, type of delivery and physical activity level had no influence on PFM pressure in postmenopausal women [13]. In addition, the correlation for maximum squeeze pressure between examiners was unaffected by age, estrogen status, size of genital hiatus, parity, BMI and degree of prolapse [12]. In using the present scale, it is important to explain that the classification is recommended for all women with the same age group as the study sample, regardless of their clinical condition.

To the authors' knowledge, this is the first study that generated a classification of manometric measurements in women regardless of age, hormonal status and obstetric factors. This makes it easier for physical therapists and patients to understand the strength degrees performed by PFM. The study by Isherwood and Rane [23] simulated a manometry classification. The authors grouped the manometry results into six categories (A to F), according to the patients’ contraction profile; however, they did not create a scale.

In recent years, manometry has been widely used as an assessment method in various clinical studies [29–32]. Manometry has proven to be an easy and quick technique for assessing PFM contraction. With the development of the manometry classification scale, the authors recommend its use for clinical and scientific purposes. In addition, the creation of a scale for a non-specific public allows the use of this data in all women.

A limitation of the proposed classification by this study concerns comparing it with other devices. In this regard, the present manometry classification scale is applicable only when using the same apparatus. The study by Barbosa et al. [33] suggests that perineometers of different brands have moderate to very low reliability, hindering comparison of results between studies with different instruments. This scale should also only be applied in clinical practice if the Peritron 9300V is used. The authors believe it is possible to use the scale with other manometry equipment; however, it will be necessary to convert the unit of measurement.

The order of PFM function assessment was not randomized for the sample. To minimize this limitation, all patients were first evaluated by bidigital palpation and then by manometry; an interval of rest was allowed between assessments. Moreover, all assessments were performed by the same physical therapist.

All degree zero patients in the modified Oxford scale were excluded from the study. The authors believe that values below 7.5 cmH_2_O in manometry may be equivalent to level 0 on the scale, assigning this value to the pressure existing in the vaginal canal with the equipment probe, which is sensitive to small compression changes.

Future studies should verify the reliability of the scale in different devices. Further studies should also validate the scale, including its application in clinical reality. In addition, studies that consider the use of the scale in different age groups or groups according to hormonal status and parity may be required.

In the present study, manometry values were stratified into a five-point scale. Using this scale, it is possible to establish pressure levels of voluntary contraction of PFM. This will help physical therapists in clinical practice to assess severity of muscle strength and to guide categorization standards in the scientific field.

## Supporting information

S1 File Evaluation form. (DOC)
