# Supplementary material for: A manometry classification to assess pelvic floor muscle function in women
Source: PLoS One. 2017 Oct 30;12(10):e0187045. doi: 10.1371/journal.pone.0187045 (PMC5662229; doi:10.1371/journal.pone.0187045)
Supplement: S1 File — (DOC) [file pone.0187045.s001.doc]

Date: _____/_____/_____

Group: _________________________

Nº _________

**“**FUNCTIONAL EVALUATION OF PELVIC FLOOR MUSCLES IN THE PHASES OFWOMEN’S LIFE”

**EVALUATION FORM**

**1. IDENTIFICATION**

Name: ____________________________________________________________Telephone:_______________________________________

Adress: _____________________________________________________________________________________________________________

**2. SOCIODEMOGRAPHIC INFORMATION**

| Occupation: ____________________________________ Birthdate: _________________ Age: _________  Marital status: ( ) Single ( ) Living common law ( ) Married ( ) Widowed ( ) Divorced/separated  Academic degrees: ( ) up tp 4 years of study ( ) 5-8 years of study ( ) 9-11 years of study ( ) over 12 years of study  Religions: ( ) christianity ( ) spiritualist ( ) Atheism ( ) other ___________________  Income: ( ) No income ( ) minimum wage ( ) 1 to 2 minimum wage ( ) 3 to 4 minimum wage ( ) > 4 wage  Family income (R$) ________________________ Number of households: ___________ |
| --- |

**3. GYNECOLOGICAL AND OBSTETRICS HISTORY**

| Gestations ( ) Yes ( ) No Number ______ Abortion ( ) Yes ( ) No Number ______  Vaginal birth ( ) Yes ( ) No Number ______ Episiotomy ( ) Yes ( ) No Number ______  Forceps delivery ( ) Yes ( ) No Number ______ Vacuum extraction ( ) Yes ( ) No Number ______  Cesarean section ( ) Yes ( ) No Number ______ |
| --- |

| Phase: reproducible ( ) transition ( ) postmenopausal ( )  Gynecological surgery ( ) Yes ( ) No  - histerectomy ( ) Yes ( ) No How long? _____________________ ( ) Partial ( ) Total  - oophorectomy ( ) Yes ( ) No How long? _____________________ ( ) Unilateral ( ) Bilateral  - perineoplasty ( ) Yes ( ) No How long? ____________________  - bladder suspension surgery ( ) Yes ( ) No How long? _______________________  - Tubal ligation surgery ( ) Yes ( ) No How long? Other/how long?____________________________ |
| --- |

**4. PHYSICAL EXAM**

| Weight ___________ Height: ___________ BMI: ___________ (weight/height^2^) |
| --- |

**5. PELVIC FLOOR MUSCLES (PFM)**

| Visible contraction ( ) Yes ( ) No Coordinated contraction ( ) Yes ( ) No Use of acessory muscles ( ) Yes ( ) No ______________________  Palpation of PFM – Oxford Scale  ( ) 0 ( ) 1 ( ) 2 ( ) 3 ( ) 4 ( ) 5  Perineometry (cmH_2_O)  Performed the exam ( ) Yes ( ) No ( ) Pain ( ) Several prolapse ( ) Other ____________________________________  1ª time _____________ 2ª time _____________ 3ª time _____________ Obs.: ______________________________________ |
| --- |

Observation:_________________________________________________________________________________________________________

Data da avaliação: _____/_____/_____

Grupo: _________________________

Nº _________

**“**AVALIAÇÃO FUNCIONAL DOS MÚSCULOS DO ASSOALHO PÉLVICO

NAS FASES DA VIDA DAS MULHERES**”**

**FICHA DE AVALIAÇÃO**

**1. IDENTIFICAÇÃO**

Nome: ____________________________________________________________Telefone:________________________________________

Endereço: ___________________________________________________________________________________________________________

**2. INFORMAÇÕES SOCIODEMOGRÁFICAS**

| Profissão/Função: ____________________________________ Data de Nascimento: _________________ Idade: _________  Estado Civil: ( ) solteira s/ união estável ( ) solteira c/ união estável ( ) casada ( ) viúva ( ) Divorciada  Grau de Instrução: ( ) analfabeta ( ) primário ( ) 1º grau ( ) 2º grau ( ) Superior Completo Sim ( ) não ( ) ________  Religião: ( ) católica ( ) evangélica ( ) espírita ( ) sem religião ( ) outro ___________________  Renda: ( ) sem renda ( ) até 1 salário mínimo ( ) 1 a 2 salários mínimos ( ) 3 - 4 salários mínimos ( ) > 4 salários  Renda familiar (R$) ________________________ No de pessoas que residem na casa: ___________ |
| --- |

**3.HISTÓRIA GINECOLÓGICA E OBSTÉTRICA**

| Gestações ( ) Sim Número ______ Aborto ( ) Sim ( ) Não Número ______  Parto Normal ( ) Sim ( ) Não Número ______ Episiotomia ( ) Sim ( ) Não Número ______  Parto a fórceps ( ) Sim ( ) Não Número ______ Parto a vácuo ( ) Sim ( ) Não Número ______  Parto Cesárea ( ) Sim ( ) Não Número ______ |
| --- |

| Fase: reprodutível ( ) transição ( ) pós-menopausa ( )  Cirurgia ginecológica ( ) Sim ( ) Não  - histerectomia ( ) Sim ( ) Não Tempo: _____________________ ( ) Parcial ( ) Total  - ooferectomia ( ) Sim ( ) Não Tempo: _____________________ ( ) Unilateral ( ) Bilateral  - perineoplastia ( ) Sim ( ) NãoTempo: ____________________ suspensão de bexiga ( ) Sim ( ) NãoTempo: _______________________  - Ligadura de trompas ( ) Sim ( ) Não Tempo: _____________________ outra/ tempo __________________________________________ |
| --- |

**4. EXAME FÍSICO**

| Peso ___________ Altura: ___________ IMC: ___________ (peso/altura^2^) |
| --- |

**5. INSPEÇÃO DA MAP**

| Contração visível ( ) Sim ( ) Não Contração coordenada ( ) Sim ( ) Não Musculatura acessória ( ) Sim ( ) Não _________________________  Palpação da MAP – Escala de Oxford  ( ) 0 ( ) 1 ( ) 2 ( ) 3 ( ) 4 ( ) 5  Perineometria (cmH_2_O)  Realizou o exame ( ) Sim ( ) Não ( ) Presença de dor ( ) Presença de prolapso ( ) Outro ____________________________________  1ª tentativa _____________ 2ª tentativa _____________ 3ª tentativa _____________ Obs: _____________ __________________________ |
| --- |

Observação:__________________________________________________________________________________________________________
